# Multipurpose Prevention Approaches with Antiretroviral-Based Formulations

**DOI:** 10.1128/AAC.02468-15

**Published:** 2016-01-29

**Authors:** Ninochka Jean-Pierre, Patrick Barnable, Larisa Kizima, Aixa Rodríguez, Samantha Seidor, Michael L. Cooney, Meredith R. Clark, Gustavo F. Doncel, Melissa Robbiani, Thomas M. Zydowsky, Natalia Teleshova, José A. Fernández-Romero

**Affiliations:** a Center for Biomedical Research, Population Council, New York, New York, USA; b CONRAD, Department of Obstetrics and Gynecology, Eastern Virginia Medical School, Arlington, Virginia, USA

## Abstract

We compared the preclinical safety and efficacy of tenofovir (TFV) 1% gel with that of MZC gel [containing 50 μM MIV-150, 14 mM Zn(O_2_CCH_3_)_2_(H_2_O)_2_, and 3% carrageenan] through a series of *in vitro*, *ex vivo*, and *in vivo* assays. The two gels showed good antiviral therapeutic indexes (50% cytotoxic concentration/50% effective concentration ratios; range, >25 to 800). MZC showed greater anti-simian-human immunodeficiency virus reverse transcriptase (SHIV-RT) activity than TFV 1% gel in rhesus macaque vaginal explants. MZC protected mice from vaginal herpes simplex virus 2 (HSV-2) challenge (*P* < 0.0001), but the TFV 1% gel did not.

## TEXT

The most recent lead microbicide formulations, tenofovir (TFV) 1% gel and the dapivirine intravaginal ring (IVR), have been investigated as means to prevent primarily human immunodeficiency virus (HIV) acquisition. Recent clinical trials like CAPRISA 004 (clinical trial registration number NCT00441298) (1, 2), conducted by the Centre for the AIDS Programme of Research in South Africa, and the VOICE trial (Vaginal and Oral Interventions to Control the Epidemic; clinical trial registration number NCT00705679) (3) have investigated the effectiveness of TFV 1% gel to prevent HIV acquisition. CAPRISA 004 was a study comparing TFV 1% vaginal gel with a placebo when used before and after sex while the VOICE trial looked at daily use of an oral tablet (TFV or Truvada) or a vaginal gel (TFV 1% gel). CAPRISA 004 and the VOICE trial showed a reduction in herpes simplex virus 2 (HSV-2) acquisition that correlated with the use of TFV vaginal gel. This was surprising since TFV has modest anti-HSV activity in cell-based assays (4), *ex vivo* explants (4), and murine models (4, 5). Herein, we explored the preclinical safety and efficacy of a dual compartment multipurpose prevention technology (MPT) microbicide gel (MZC, which contains 50 μM MIV-150, 14 mM zinc acetate dihydrate, and 3% carrageenan [CG]) targeting HIV, HSV, and human papillomavirus (HPV) (6) in a side-by-side comparison with the TFV 1% gel. A recently completed phase 3 trial (FACTS 001; clinical trial registration number NCT01386294) of TFV 1% gel was designed that followed the CAPRISA 004 dosing strategy but had a larger number of participants. The gel was not proven to be effective and poor user adherence to the dosing regimen likely contributed to the outcome of the trial. A reduced glycerin version of this gel is currently in a phase 2 trial as a potential rectal microbicide (7).

MZC, TFV 1%, and 3% CG gels were formulated as previously described (1, 6). MZC, like TFV 1% gel, is a clear and semisolid formulation. However, based on physicochemical properties, the formulations differ mainly in pH (TFV 1%, 5.0; MZC, 6.9) and osmolality (TFV 1% gel, 3,358 mosmol/kg; MZC gel, 447 mosmol/kg). The MZC gel contains only 0.002% of the non-nucleoside reverse transcriptase inhibitor (NNRTI) MIV-150 versus 1% of the NRTI TFV in the 1% gel.

Antiviral activity against HIV-1 was tested using the standardized TZM-bl-based assay (6) or the peripheral blood mononuclear cell (PBMC)-based assay (6). Briefly, TZM-bl cells (1.5 × 10^5^/ml) or activated PBMCs (2 × 10^6^/ml) were treated for 1 h with dilutions of gels (triplicates) before adding 100 focus forming units (FFU) or one hundred 50% tissue culture infective doses (TCID_50_) of virus, respectively. TZM-bl cells were incubated for 72 h before staining with 5-bromo-4-chloro-3-indolyl-β-d-galactopyranoside (X-Gal) to count FFU. The supernatant was replaced for PBMCs with fresh stimulation medium on days 1 and 4 postinfection. The p24 level in the supernatant was determined on day 7 after infection by p24 enzyme-linked immunosorbent assay (ELISA) (ZeptoMetrix, Buffalo, NY). The 50% cytotoxic concentration (CC_50_ values) of each gel formulation was estimated using XTT and CyQuant by running the antiviral assay in the absence of virus (6). The 50% effective concentrations (EC_50_s) were calculated based on gel dilution factor in order to compare the efficacies of the two gels, each containing a different active pharmaceutical ingredient (API). By comparing the EC_50_s based on gel dilution, we observed how MZC with only 0.002% of MIV-150 can achieve better or similar antiviral activity than TFV gel containing 1% of the API. MZC gel was generally more potent than TFV 1% gel in blocking HIV-1 infection in TZM-bl or PBMC with the clear exception of one multidrug-resistant (MDR) strain [OL-1/4(II)d4] containing 2 mutations (K101E and Y181I) associated with decreased susceptibility of viruses to NNRTI (Table 1). Similarly, TFV 1% gel showed an increase in EC_50_s for two strains (71361-1 and 56252-1) containing the 65R amino acid change associated with HIV resistance to TFV. Although NNRTIs are known to select resistant viruses rapidly, MIV-150 seems to select resistance at a slower pace compared to other NNRTIs and requires two or more mutations in a single genome to decrease HIV susceptibility (8). Additionally, it is important to mention that resistance development in topically applied antiretrovirals is not yet fully understood.

**TABLE 1:** Antiviral activity of MZC and 1% TFV gels against HIV-1

HIV-1^*a*^ strain	EC_50_ based on gel dilution factor (95% confidence interval)^*b*^ for:		TI^*c*^ for:	
	MZC^*d*^	1% TFV	MZC	1% TFV
BaL^*e*^^,^^*f*^	3 × 10^−5^ (2 × 10^−5^ to 4 × 10^−5^)	3.7 × 10^−4^ (3 × 10^−4^ to 5 × 10^−4^)	>133	>10
ADA-M^*e*^^,^^*f*^	2 × 10^−5^ (1 × 10^−5^ to 3 × 10^−5^)	2.2 × 10^−4^ (1 × 10^−4^ to 3 × 10^−4^)	>200	>18
MN^*e*^^,^^*f*^	0.4 × 10^−5^ (0.3 × 10^−5^ to 0.6 × 10^−5^)	3.2 × 10^−4^ (1 × 10^−4^ to 7 × 10^−4^)	>1,000	>12
MG505.WOM.ENV.CZ^*g*^	2 × 10^−5^ (1 × 10^−5^ to 4 × 10^−5^)	6 × 10^−5^ (3 × 10^−5^ to 1 × 10^−4^)	>200	>66
NL4-3^*f*^	2 × 10^−4^ (8 × 10^−5^ to 7 × 10^−4^)	4 × 10^−4^ (2 × 10^−4^ to 7 × 10^−4^)	>250	>125
92UG029^*g*^	4 × 10^−4^ (3 × 10^−4^ to 5 × 10^−4^)	3 × 10^−4^ (1 × 10^−4^ to 1 × 10^−3^)	>125	>166.7
91US056^*f*^	4 × 10^−4^ (2 × 10^−4^ to 7 × 10^−4^)	3 × 10^−4^ (1.5 × 10^−4^ to 7 × 10^−4^)	>125	>166.7
92BR014^*f*^	3 × 10^−4^ (1 × 10^−4^ to 7 × 10^−4^)	1 × 10^−4^ (5 × 10^−5^ to 3 × 10^−4^)	>166.7	>500
92HT593^*f*^	1 × 10^−4^ (5 × 10^−5^ to 2 × 10^−4^)	1 × 10^−4^ (6 × 10^−5^ to 2 × 10^−4^)	>500	>500
97ZA009^*h*^	1 × 10^−4^ (9 × 10^−5^ to 2 × 10^−4^)	5 × 10^−4^ (2 × 10^−4^ to 1 × 10^−3^)	>500	>100
97USNG30^*h*^	6 × 10^−4^ (5 × 10^−4^ to 9 × 10^−4^)	5 × 10^−4^ (3 × 10^−4^ to 7 × 10^−4^)	>83.3	>100
96USNG31^*h*^	6 × 10^−4^ (2 × 10^−4^ to 3 × 10^−3^)	4 × 10^−4^ (1 × 10^−4^ to 9 × 10^−4^)	>83.3	>125
CMU06^*i*^	1 × 10^−4^ (1 × 10^−5^ to 2 × 10^−4^)	1 × 10^−4^ (7 × 10^−5^ to 4 × 10^−4^)	>500	>500
92TH020^*i*^	6 × 10^−5^ (4 × 10^−5^ to 1 × 10^−4^)	1 × 10^−4^ (7 × 10^−5^ to 3 × 10^−4^)	>833.3	>500
93TH051^*i*^	4 × 10^−4^ (2 × 10^−4^ to 1 × 10^−3^)	3 × 10^−4^ (1 × 10^−4^ to 6 × 10^−4^)	>125	>166.7
35764-2^*f*^	8 × 10^−5^ (3 × 10^−5^ to 2 × 10^−4^)	4 × 10^−4^ (2 × 10^−4^ to 1 × 10^−3^)	>625	>125
7295-1^*f*^	5 × 10^−4^ (4 × 10^−4^ to 7 × 10^−4^)	6 × 10^−4^ (4 × 10^−4^ to 8 × 10^−4^)	>100	>83.3
29129-2^*f*^	2 × 10^−4^ (9 × 10^−5^ to 3 × 10^−4^)	7 × 10^−4^ (5 × 10^−4^ to 1.1 × 10^−3^)	>250	>71.4
56252-1^*f*^	5 × 10^−4^ (2 × 10^−4^ to 1.4 × 10^−3^)	∼1 × 10^−2^	>100	>5
4755-5^*f*^	2 × 10^−4^ (1 × 10^−4^ to 5 × 10^−4^)	∼3 × 10^−4^	>250	>166.7
1617-1^*f*^	1 × 10^−4^ (6 × 10^−5^ to 4 × 10^−4^)	2 × 10^−3^ (5 × 10^−4^ to 5 × 10^−3^)	>500	>25
7324-4^*f*^	3 × 10^−4^ (2 × 10^−4^ to 5 × 10^−4^)	4 × 10^−4^ (1 × 10^−4^ to 1.3 × 10^−3^)	>166.7	>125
7324-1^*f*^	1 × 10^−4^ (7 × 10^−5^ to 3 × 10^−4^)	4 × 10^−4^ (2 × 10^−4^ to 4 × 10^−4^)	>500	>166.7
8415-2^*f*^	4 × 10^−4^ (1 × 10^−4^ to 1.2 × 10^−3^)	5 × 10^−4^ (2 × 10^−4^ to 1.1 × 10^−3^)	>125	>100
6463-13^*f*^	4 × 10^−5^ (2 × 10^−5^ to 1 × 10^−4^)	3 × 10^−4^ (1 × 10^−4^ to 7 × 10^−4^)	>1,250	>166.7
7136-1^*f*^	3 × 10^−4^ (1 × 10^−4^ to 9 × 10^−4^)	∼1 × 10^−3^	>1,66.7	>50
V16770-2^*f*^	5 × 10^−4^ (4 × 10^−4^ to 7 × 10^−4^)	5 × 10^−4^ (2 × 10^−4^ to 1.3 × 10^−3^)	>100	>100
V17763-5^*f*^	4 × 10^−4^ (1 × 10^−4^ to 1.6 × 10^−3^)	2 × 10^−4^ (2 × 10^−5^ to 1.3 × 10^−3^)	>125	>250
W1023892-2^*f*^	2 × 10^−4^ (6 × 10^−5^ to 5 × 10^−4^)	1 × 10^−4^ (3 × 10^−5^ to 2 × 10^−4^)	>250	>500
J18-1(2)^*f*^	2 × 10^−4^ (1 × 10^−4^ to 7 × 10^−4^)	3 × 10^−4^ (1 × 10^−4^ to 8 × 10^−4^)	>250	>166.7
OL-1/4(II)d4^*f*^	>2.5 × 10^−3^	3 × 10^−4^ (1 × 10^−4^ to 7 × 10^−4^)	ND^*j*^	>166.7
C18-15d7^*f*^	1.5 × 10^−3^ (1.1 × 10^−3^ to 1.9 × 10^−3^)	1 × 10^−3^ (4 × 10^−4^ to 2 × 10^−3^)	>33.3	>50

a The HIV-1_MN_, HIV-1_ADA-M_, and HIV-1_BaL_ laboratory strains were provided by J. D. Lifson at the AIDS and Cancer Virus Program, Leidos Biomedical Research, Inc. HIV-1 transmitted/founder virus, clone MG505.WOM.ENV.CZ, was provided by James Arthos at NIAID, NIH (Bethesda, MD, USA). Additionally, a panel of 28 viruses/clones (6) representing different HIV-1 clades and multidrug-resistant (MDR) strains were tested.

b EC_50_s in TZM-bl and PBMC were calculated using a dose-response-inhibition analysis on GraphPad Prism v5.0c software (GraphPad Software, San Diego, CA). The EC50s are based on the gel dilution factor.

c Therapeutic indexes (TI = CC_50_/EC_50_). CC_50_ was >5E−02 in PBMCs and >4E−03 in TZM-bl. CC50s are based on the gel dilution factor.

d Published data (4).

e Cytotoxicity and antiviral assays were performed in TZM-bl cells, and PBMCs were used with all other viruses.

f Clade B.

g Clade A.

h Clade C.

i Clade E.

j ND, not determined.

Cell-based assays are excellent tools for screening potential microbicides, for testing antiviral properties against a variety of isolates/MDR strains, and for monitoring the stability of formulations. However, the testing of a lead formulation in the explant model allows for assessment of preclinical safety and efficacy in a more relevant HIV target cell and architectural context. We tested MZC and TFV 1% gel in our *ex vivo* rhesus macaque (RM) vaginal explant model using cell-free virus inoculum and also cell-associated virus.

**FIG 1:**
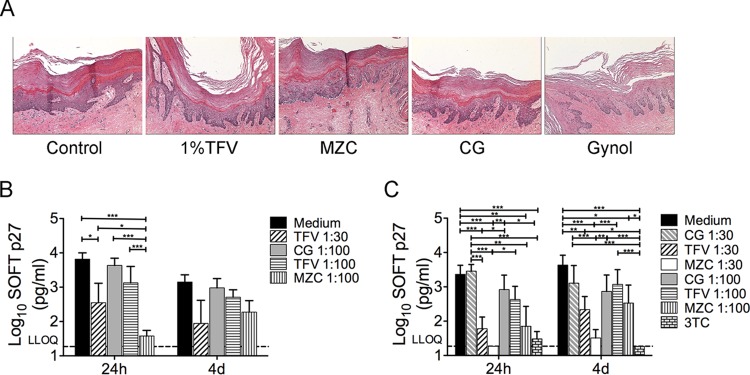
MZC and TFV gels have no apparent effect on tissue architecture and reduce cell-free and cell-associated SHIV-RT infection of macaque vaginal explants. (A) MZC and 1% TFV gels do not induce histopathological changes in macaque vaginal tissues using the previously described polarized macaque vaginal explant model (9). Results representative of 3 experiments are shown at 10× magnification. (B) Tissue challenge with cell-free SHIV-RT infection was performed as described in the study by Barnable et al. (9). Briefly, explants were immersed in diluted gels (1:30 or 1:100) (versus untreated controls) for 18 h in the presence of PHA/IL-2. Then tissues were washed and challenged with SHIV-RT (10^4^ TCID_50_) 24 h or 4 days after exposure to the gels. The tissues were washed again 18 h after virus challenge and cultured for 14 days in the presence of IL-2. SIV p27 levels were measured over the culture period. (C) Tissue challenge with cell-associated SHIV-RT was performed as previously described (9). Briefly, explants were immersed in medium containing diluted gels (versus untreated controls) for 18 h in the presence of PHA/IL-2. Then tissues were washed and challenged (18 h) with mitomycin-C-treated, SHIV-RT-infected PBMCs (10^3^ infected PBMCs/27 TCID_50_ per explant; 2 to 4 replicates) 24 h or 4 days after exposure to the gels. Then tissues were washed and cultured for 14 days in the presence of IL-2 (versus 10 μM 3TC control) and analyzed (results shown in panel B). No released p27 was detected in cultures of control mitomycin-C-treated, SHIV-RT-infected PBMCs cultured alone (not shown). The analysis was done using a log-normal generalized linear mixed model with the individual replicate data. (B, C) Shown are results from SOFT analyses (mean ± standard error of the mean [SEM]) of *n* = 5 to 9 (24 h) and *n* = 3 to 9 (4 days) experiments. SHIV-RT p27 concentrations of individual replicate values more than or equal to the lower limit of quantification (LLOQ) were assumed to be log-normal. Type 3 *F* tests were used to determine the overall effect of treatment. Tukey's adjusted *t* tests were used for pairwise comparisons of treatments. The analysis was performed with SAS v9.4 and SAS/STAT v13.1 with α = 0.05. *, *P* values < 0.05; **, *P* values < 0.01; and ***, *P* values < 0.001 for relevant comparisons. MZC gel was tested only at 1:100 dilution in the cell-free model due to tissue availability.

Macaque vaginal mucosal samples (biopsy specimens or necropsy tissues) were collected and transported overnight as previously described (9). Histological analysis was performed on polarized macaque vaginal tissue explants after overnight treatment (∼18 h) with neat MZC, TFV 1%, or CG gels following the procedure described in Barnable et al. (9). Neither gel induced vaginal epithelial damage (Fig. 1A) or decreased viability—based on 3-(4,5-dimethyl-2-thiazolyl)-2,5-diphenyl-2H-tetrazolium bromide (MTT) assay results (data not shown). The antiviral activity of diluted MZC, CG (1:100), or TFV 1% (1: 30 or 1:100) gels against *ex vivo* cell-free simian-human immunodeficiency virus reverse transcriptase (SHIV-RT) challenge was performed as described in Barnable et al. (9) in an immersion culture model. Briefly, explants were immersed overnight (∼18 h) in 1:30 and 1:100 diluted MZC, 1% TFV, or CG (versus untreated controls) in the presence of phytohemagglutinin (PHA)/interleukin-2 (IL-2). The diluted gels did not decrease tissue viability (MTT data not shown). After exposure to the gels, the explants were washed and then challenged with SHIV-RT (10^4^ TCID_50_/explant) 24 h or 4 days after gel exposure. Following 18 h of viral challenge, the tissues were washed and cultured for 14 days in the presence of IL-2. Simian immunodeficiency virus (SIV) p27 release was measured at 0, 3, 7, 11, and 14 days of culture by ELISA. Cell-associated SHIV-RT infection was performed following a different model (10) where macaque vaginal explants were immersed overnight in medium containing diluted MZC, 1% TFV, or CG gels (versus untreated controls). The samples were then washed and cultured for 24 h or 4 days before being challenged with mitomycin-C-treated, SHIV-RT-infected PBMC (10^3^ infected PBMC/27 TCID_50_ per explant; 2 to 4 replicates) for ∼18 h, washed, and maintained in culture medium in the presence of IL-2 (100 U/ml) (versus 10 μM lamivudine [3TC] control). Mitomycin-C-treated, SHIV-RT-infected PBMCs cultured alone were included as controls. The SHIV-RT p27 concentration was determined in supernatants during 14-day culture. Soft endpoint (SOFT) analysis demonstrated that MZC and TFV 1% gels significantly reduce SHIV-RT infection from cell free (Fig. 1B) and cell-associated (Fig. 1C) challenges, with MZC being more effective against cell-free virus 24 h after diluted gel (1:100) application (*P* < 0.0001). Similar results were observed with cumulative analysis (not shown).

**FIG 2:**
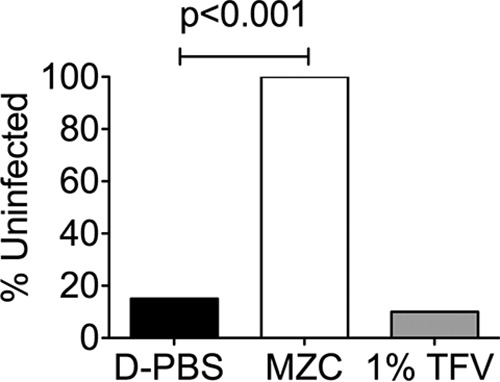
MZC, but not 1% TFV gel, protects mice from HSV-2 vaginal challenge. Depo-Provera-treated BALB/c mice were treated intravaginally with 10 μl of the indicated formulations 1 h before and after HSV-2 challenge with 5 × 10^3^ PFU (*n* = 20/formulation). The percentages of uninfected animals are shown for each treatment group. Fisher's exact test was used for statistical comparison, and *P* values of <0.05 were taken as statistically significant.

We have previously shown that the combination of CG and zinc acetate (as in the MZC formulation) results in antiviral synergy (*in vitro* and *in vivo*) against HSV-2 (11). We explored the anti-HSV-2 activity of MZC and of TFV 1% gels in a murine model. Depo-Provera-treated BALB/c mice were dosed with 10 μl of test gel intravaginally 1 h prior to HSV-2 infection plus 1 h after HSV-2 infection to mimic the BAT24 dosing strategy used in the CAPRISA 004 and FACTS 001 trials (BAT24 refers to one dose of gel before sex and a second dose of gel as soon as possible after sex and no more than two doses in a 24-hour period) (12). Mice were challenged with 10 μl of HSV-2 G (5 × 10^3^ PFU/mouse) and were examined and scored daily for 21 days as previously described (11). Despite being tested in a less-stringent murine model (lower virus inoculum compare to previous evaluation in this model [6, 11]), the TFV 1% gel did not protect mice from HSV-2 infection while the MZC gel protected 100% of the animals (Fig. 2).

A possible explanation for these divergent results (compared to CAPRISA 004 results in humans) is that TFV phosphorylation and/or TFV uptake may be less efficient in mice than in human cells. In fact, subtherapeutic (below a lower limit of quantification [LLOQ] of <100 ng/g) TFV diphosphate (TFV-DP) levels were found in murine cervicovaginal tissue, and even TFV-only levels were low (median, 5400 ng/g) as determined by liquid chromatography-tandem mass spectrometry (LC-MS/MS) (13).

MZC's potent *in vitro* and *in vivo* anti-HPV activities make this formulation a very appealing MPT candidate targeting three noncurable viral sexually transmitted infections (STIs) (6). However, poor adherence in clinical trials is an important issue that has overshadowed the success of microbicide gels in the HIV preexposure prophylaxis field. In light of this, the MZC combination may be explored not only as a gel (with potential for a rectal microbicide) but also as an IVR that incorporates levonorgestrel (LNG) to prevent unintended pregnancy (14) (a similar approach is being tested in a phase 1 trial with a TFV + LNG IVR [ClinicalTrials registration no. NCT02235662] [15]). Importantly, the results shown in this paper provide information about API levels that need to be released from alternative delivery systems (e.g., IVR) in order to be safe and achieve protection against HIV infection. Adding a contraceptive, targeting more than one STI, and providing different choices for drug delivery may increase demand/uptake as well as the efficiencies of delivery and access. The MZC combination is a promising MPT that was successfully evaluated in a phase 1 trial (Population Council 558; clinical trial registration number NCT02033109), and the results shown herein support moving forward with its clinical evaluation.
